# Progress in Disease of Digestive Organs

**Published:** 1902-04-05

**Authors:** 


### Progress in Disease of Digestive Organs.

(*Continued from page* 438.)

**Splashing Sounds** in the stomach must be distinguished from "murmurs." The latter are formed when gas and little or no fluid is present, and are of no diagnostic significance. Splashing sounds are of importance only if the fluid has been some time in the stomach. Thus if present during digestion they point to atony, if present after this period they indicate motor insufficiency (Kuttner[8]).

**Phlegmonous Gastritis.**—W. Cayley[9] records an instance in a woman marked by rigors, vomiting, abdominal pain, and distension, with dilatation of the stomach. Post-mortem there was found purulent infiltration of all the coats of the stomach between the mucous and peritoneal ones, with streptococci and B. coli. Such infection generally follows intemperance, injury, or errors of diet. There may be vomiting of pus. Pneumococcal gastritis is marked by septicæmia with petechiæ and a fibrinous exudation over the surface of the stomach. Rolleston refers also to the duodenal ulcers found in pneumonia, but these may be of toxic origin as the cocci have not been found in them. O. A. Schultze[10] gives a detailed report of the examination of a stomach affected with phlegmonous gastritis; streptococci were also found here in the submucosa and muscular coats. The stomach was not dilated. The patient, who was insane, suffered from vomiting and abdominal pain for a few days before death. Another case was reported by L. A. Conner with the same violent gastric symptoms during the last days of his life.

**Gastric Cancer.**—Hemmeter[11] notices that an early diagnosis can often be made if regular analyses of the stomach contents are carried out. There is usually a latent period of three or four months in which there are no symptoms but those of chronic gastritis with progressive loss of weight. There is nothing absolutely distinctive in the age of the patients, though they are usually over 40, nor in the dyspeptic symptoms. The absence of acid depends on whether or no the cancer affects the middle portion of the stomach, since the pylorus does not share in the secretion. Excess of lactic acid, if to the extent shown by Uffelman's test, is of some value, since it means absence of H.Cl. and of absorption, stagnation and loss of digestive ferments, which are common in cancer. When a soft tube has been passed along the walls fragments of epithelium can be often washed out, and if these show cells containing karyokinetic figures, an early diagnosis may be made. The Oppler-Boas bacillus should also be looked for, and the reduction of proteid digestion to under 30 per cent. He discusses many other symptoms, but finds them of doubtful utility, and finally decides that in really early stages of the disease the order of importance of the symptoms is (*a*) chronic gastritis getting worse after four weeks' proper treatment, (*b*) progressive loss of motility, (*c*) progressive loss of H.Cl. concurrently with *b*, (*d*) the presence of many atypical mitoses in the cells removed after gastric "curettage."

[8] Med. Rec., Jan. 11. [9] Lancet Jan. 25. [10] Med. Rec., Nov. 30. [11] Lancet, Sept. 28.

